# Sustained corneal nerve loss predicts the development of diabetic neuropathy in type 2 diabetes

**DOI:** 10.3389/fnins.2024.1393105

**Published:** 2024-07-02

**Authors:** Georgios Ponirakis, Ibrahim Al-Janahi, Einas Elgassim, Moayad Homssi, Ioannis N. Petropoulos, Hoda Gad, Adnan Khan, Hadeel B. Zaghloul, Hamda Ali, Mashhood A. Siddique, Fatima F. S. Mohamed, Lina H. M. Ahmed, Youssra Dakroury, Abeer M. M. El Shewehy, Ruba Saeid, Fadwa Mahjoub, Shaikha N. Al-Thani, Farheen Ahmed, Rawan Hussein, Salah Mahmoud, Nebras H. Hadid, Aisha Al Obaidan, Iuliia Salivon, Ziyad R. Mahfoud, Mahmoud A. Zirie, Yousuf Al-Ansari, Stephen L. Atkin, Rayaz A. Malik

**Affiliations:** ^1^Department of Medicine, Weill Cornell Medicine-Qatar, Qatar Foundation, Doha, Qatar; ^2^National Diabetes Center, Hamad General Hospital, Hamad Medical Corporation, Doha, Qatar; ^3^Royal College of Surgeons in Ireland Bahrain, Adliya, Bahrain; ^4^Division of Cardiovascular Sciences, Faculty of Biology, Medicine and Health, University of Manchester, Manchester, United Kingdom; ^5^Faculty of Science and Engineering, Manchester Metropolitan University, Manchester, United Kingdom

**Keywords:** sustained corneal nerve damage, corneal confocal microscopy, type 2 diabetes, risk factor reduction, predictive biomarker, diabetic neuropathy

## Abstract

**Introduction:**

This study was undertaken to investigate whether sustained rather than a single measure of corneal nerve loss was associated with the onset of diabetic peripheral neuropathy (DPN) and the progression of neuropathic symptoms and deficits in individuals with type 2 diabetes (T2D).

**Methods:**

Participants underwent clinical, metabolic testing and assessment of neuropathic symptoms, vibration perception threshold (VPT), sudomotor function, and corneal confocal microscopy (CCM) at baseline, 1, 2, and 4–7 years. Sustained corneal nerve loss was defined as abnormal corneal nerve fiber density (CNFD, <24 fibers/mm^2^), corneal nerve branch density (CNBD, <21 branches/mm^2^), and corneal nerve fiber length (CNFL, <16 mm/mm^2^) persisting for ≥50% of the study duration.

**Results:**

A total of 107 participants with a mean duration of T2D of 13.3 ± 7.3 years, aged 54.8 ± 8.5 years, underwent baseline and follow-up assessments over a median duration of 4 years, ranging from 1 to 7 years. The DPN prevalence at baseline was 18/107 (16.8%), and of the 89 participants without DPN at baseline, 13 (14.6%) developed DPN during follow-up. Approximately half of the cohort had sustained corneal nerve damage, and corneal nerve measures were significantly lower in this group than those without sustained damage (*p* < 0.0001). Sustained corneal nerve damage was associated with the development of DPN (*p* < 0.0001), a progressive loss of vibration perception (*p* ≤ 0.05), an increased incidence of burning pain, numbness, or a combination of both (*p* = 0.01–0.001), and a borderline association with progressive sudomotor dysfunction (*p* = 0.07). Sustained abnormal CNFL effectively distinguished between participants who developed DPN and those who did not (AUC: 76.3, 95% CI: 65.9–86.8%, *p* < 0.0001), while baseline and other sustained measures did not predict DPN onset.

**Conclusion:**

Sustained abnormal CCM is associated with more severe corneal nerve damage, DPN development, and the progression of neuropathic symptoms and deficits. Regular CCM monitoring may enable the identification of those at greater risk of developing and worsening DPN who may benefit from more aggressive risk factor reduction.

## 1 Introduction

Diabetic peripheral neuropathy (DPN) affects at least 50% of people with diabetes and is associated with pain, numbness, loss of sensation, and diabetic foot ulceration (Pop-Busui et al., 2017). Multiple risk factors, including hyperglycemia, hypertension, hyperlipidemia, increased weight, and reduced physical activity, play a significant role in the development and progression of DPN. The identification of patients at the highest risk for the development and progression of DPN would enable the selection of patients for more aggressive risk factor reduction and allow the identification of individuals who may benefit the most in clinical trials of disease-modifying therapies.

Symptoms and signs, as well as quantitative sensory tests, are highly variable (Werner et al., 2013). While nerve conduction studies (Mallik and Weir, 2005) and skin biopsies are sensitive measures of nerve damage, they require expertise to acquire and interpret and have limited utility, especially over time (Guldiken et al., 2023). Corneal confocal microscopy (CCM), a rapid, non-invasive measure of small nerve fiber damage and repair, has emerged as a predictive biomarker for the development of DPN (Lewis et al., 2020; Perkins et al., 2021). Studies have established the predictive accuracy of CCM for incident DPN (Pritchard et al., 2015; Perkins et al., 2021). However, relying on a single-time-point measurement of corneal nerve fiber length (CNFL) may have limitations as the trajectory of DPN and the change in corneal nerve morphology are not linear and are influenced by multiple risk factors such as HbA1c, lipid profile, body weight, physical activity, and ongoing treatment (Ponirakis et al., 2020, 2022).

This prospective longitudinal study investigated whether sustained corneal nerve damage is associated with incident DPN and the progression of neuropathic symptoms and deficits in individuals with type 2 diabetes (T2D). It also evaluated the predictive ability of single-time-point baseline CCM measurements and sustained abnormal CCM measures for DPN onset.

## 2 Materials and methods

### 2.1 Project design

Participants with type 2 diabetes (T2D) and healthy controls aged between 18 and 80 years were recruited from the National Diabetes Center in Hamad General Hospital in Qatar between January 2017 and October 2022. The study obtained ethics approval from the WCM-Q IRB (#14–00058 and 20–00024) and HMC IRB (#15103/15 and MRC-01-21-386) and adhered to the principles of the Declaration of Helsinki. All participants provided informed consent prior to enrollment.

The exclusion criteria for all participants included a history of allergy to oxybuprocaine, a local anesthetic used for the CCM, severe chronic dry eyes and corneal dystrophies, and a history of ocular injury or surgery in the preceding 12 months. The exclusion criteria also included chronic kidney disease (CKD) stages 4 and 5, medication leading to insulin resistance (e.g., corticosteroids), pregnancy, active retinopathy, and any cause of neuropathy other than diabetes, including Sjogren’s syndrome, systemic lupus erythematosus, human immunodeficiency virus (HIV), hepatitis B and C, inherited neuropathies, tumors, and alcohol excess. Healthy controls did not have chronic dry eyes, corneal dystrophies, a history of ocular injury or surgery in the preceding 12 months, or other causes of neuropathy, such as diabetes, Sjogren’s syndrome, systemic lupus erythematosus, HIV, hepatitis B and C, inherited neuropathies, tumors, or excessive alcohol consumption.

Participants with T2D were assessed at baseline and years 1, 2, and between 4 and 7 years. Healthy controls were enrolled only for the baseline visit to define the cutoff values for abnormal corneal nerve morphology.

### 2.2 Independent variables

Corneal nerve morphology was quantified using the Heidelberg Retina Tomograph and Rostock Cornea Module (Heidelberg Engineering GmbH, Heidelberg, Germany) (Petropoulos et al., 2013). The participants’ eyes were anesthetized using a drop of oxybuprocaine hydrochloride 0.4% (Chauvin Pharmaceuticals, Chefaro, United Kingdom). Viscotears gel (Carbomer 980, 0.2%, Novartis, UK) was applied on the front of the eye as the coupling agent between the cornea and the cap on the CCM. The participant was instructed to fixate on a target with the eye not being examined. Several scans of the sub-basal nerve plexus in the central cornea were captured per eye for ~2 min. The field of view of each image is 400×400 μm. At a separate time, three high-clarity images per eye of the sub-basal nerve plexus were selected by one researcher blind to the participant’s health condition. Criteria for image selection included depth, focus position, and contrast (Kalteniece et al., 2017). CNFD (number of main nerve fibers/mm^2^), CNBD (number of branches/mm^2^), and CNFL (length of main nerves and branches mm/mm^2^) were manually quantified using CCMetrics (Dabbah et al., 2011).

Abnormal CNFD, CNBD, and CNFL were based on a cutoff value of 1.5 standard deviations (SDs) below the mean of age-matched healthy controls and were considered sustained when persisting for ≥50% of the study duration.

### 2.3 Dependent variables

Neuropathic symptoms were assessed using the Douleur Neuropathique en 4 (DN4) questionnaire (Spallone et al., 2012). The DN4 comprises questions related to neuropathic symptoms: burning, painful cold, electric shocks, tingling, pins and needles, numbness, and itching.

Vibration perception threshold (VPT) was measured on the pulp of the large toe with a Neurothesiometer (Horwell, Scientific Laboratory Supplies, Wilford, Nottingham, United Kingdom). The test was repeated three times, and the average value was recorded.

Sudomotor nerve function was quantified by evaluating electrochemical skin conductance (ESC) using Sudoscan (Impeto Medical SAS) (Selvarajah et al., 2015). Sudoscan evaluates sympathetic innervation of the sweat gland based on sweat chloride concentrations in response to the voltage applied and is reported as ESC in microsiemens (μS).

The diagnosis of DPN was based on either ≥4 neuropathic symptoms and impaired VPT ≥15 V in the feet or ≥ 4 neuropathic symptoms and abnormal CNFL ≤17 mm/mm^2^.

### 2.4 Covariates

Clinical and metabolic measures, including age, diabetes duration, body mass index (BMI), systolic (SBP) and diastolic (DBP) blood pressure, HbA1c, and lipid profile, were recorded from the electronic medical record system.

### 2.5 Data analysis and statistics

This is the first prospective study investigating whether sustained abnormal CCM measures are associated with the development of DPN; therefore, no power calculation was determined.

Numeric variables and frequency distributions for categorical variables were summarized as mean ± standard deviation or n (%). Continuous and categorical variables between the groups with sustained and non-sustained abnormal corneal nerve measures were compared using the unpaired *t*-test and chi-square, respectively.

A bivariate linear regression analysis was performed with VPT, ΔVPT, DN4 score, ΔDN4 score, ESC, and ΔESC as dependent variables, sustained abnormal CNFD, CNBD, and CNFL as independent variables, and age, diabetes duration, blood pressure, body weight, BMI, HbA1c, and lipid profile as confounders. All dependent variables were normally distributed, as assessed by Q–Q plots and histograms. Dependent variables that were significant at the bivariate level were included in the multiple linear regression analysis and presented as the regression coefficient (95% CI).

A binary logistic regression analysis was performed with the development of DPN and burning pain and/or numbness as dependent variables, sustained abnormal CNFD, CNBD, and CNFL as independent variables, and age, diabetes duration, poor glycemic control, hyperlipidemia, hypertension, and obesity as confounders. Variables with a *p*-value of ≤0.05 at the bivariate level were included in the multiple logistic regression. Adjusted odds ratios (AORs), 95% confidence intervals (CIs), and *p*-values are presented.

A receiver operating characteristic (ROC) curve analysis was used to determine the predictive utility of abnormal baseline and sustained abnormal CCM measures for the onset of DPN. The area under the curve (AUC), 95% CI, and p-value were calculated to quantify the effectiveness of these measures in discriminating between participants with and without the development of DPN during the study.

All statistical calculations were performed using IBM-SPSS version 26 (SPSS Inc., Armonk, NY). A two-tailed p-value of ≤0.05 was considered significant.

## 3 Results

Of the 134 participants with T2D enrolled, 107 (79.9%) aged 54.8 ± 8.5 years old, with a mean duration of T2D of 13.3 ± 7.3 years, underwent follow-up assessments. Of the 107 participants, 29 (27.1%) completed 1 follow-up assessment, 38 (35.5%) completed 2 follow-up assessments, and 40 (37.4%) completed all 3 follow-up assessments. The median duration of follow-up assessments was 4 years, ranging from 1 to 7 years.

The cutoff values for abnormal CNFD, CNBD, and CNFL based on 1.5 standard deviations below the mean of 57 closely age-matched healthy controls (50.6 ± 16.9 vs. 54.8 ± 8.5 years, *p* = 0.11) were ≤ 24 fibers/mm^2^, ≤21 branches/mm^2^, and ≤ 16 mm/mm^2^, respectively.

Over a median duration of 4 years, sustained abnormal CNFD, CNBD, and CNFL were present in 51 (48%), 23 (22%), and 63 (59%) of the T2D cohort, respectively. Within the remaining cohort, the majority had normal corneal nerve morphology (85.2–91.1%) throughout the study duration, while a proportion had abnormal corneal nerve morphology on one occasion only (temporary) (8.9–14.8%).

**Figure 1:**
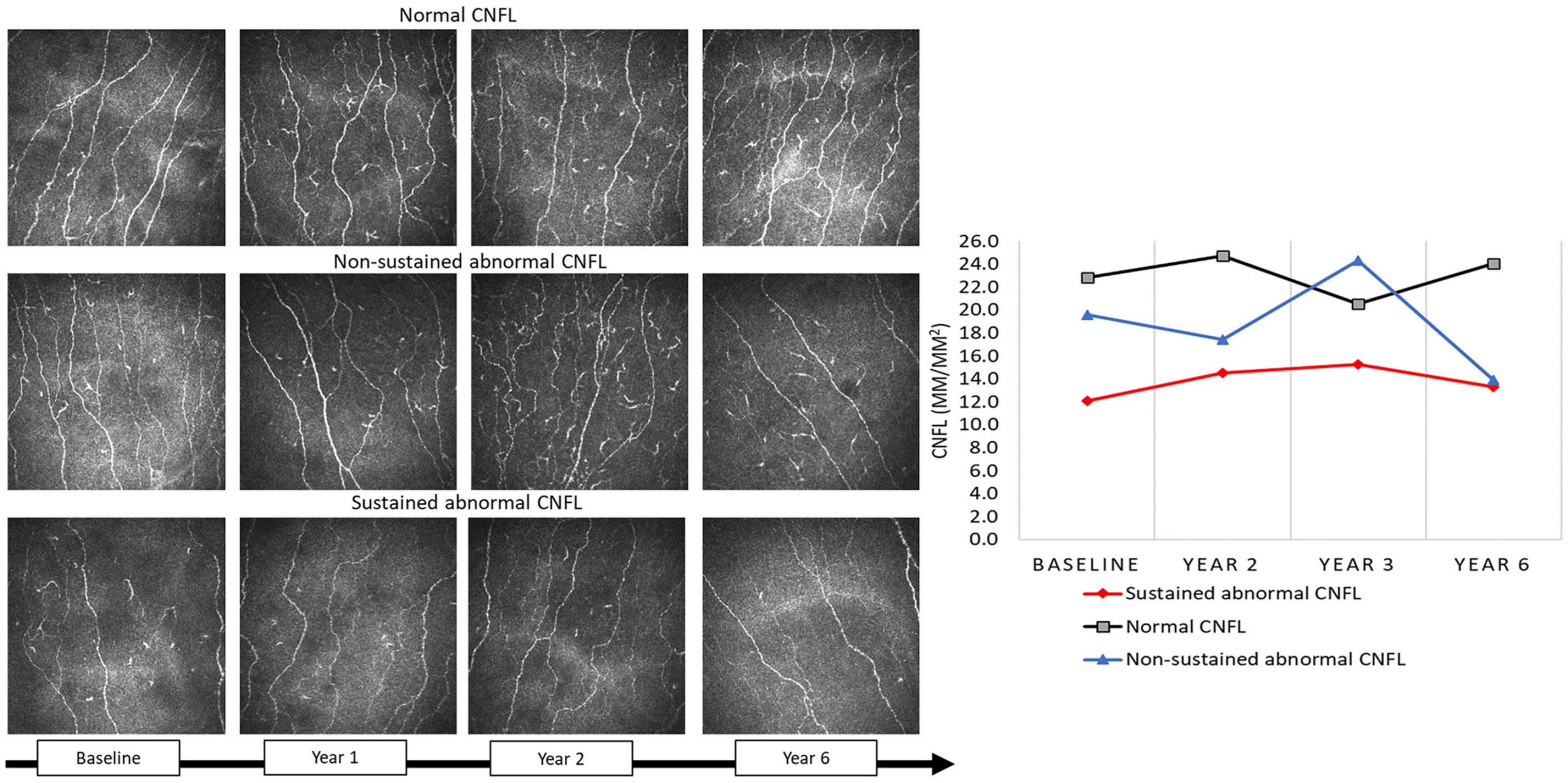
CCM images in a participant with type 2 diabetes with normal CNFL, non-sustained abnormal CNFL, and sustained abnormal CNFL at baseline, 1, 2, and year 6.

Corneal nerve measures differed significantly between those with normal, temporary, and sustained abnormality in corneal nerve morphology: CNFD 32.3 ± 6.6 fibers/mm^2^ vs. 28.2 ± 3.5 fibers/mm^2^ vs. 21.3 ± 7.6 fibers/mm^2^ (*p* < 0.0001); CNBD 78.8 ± 31.4 branches/mm^2^ vs. 61.8 ± 41.4 branches/mm^2^ vs. 32.0 ± 28.6 branches/mm^2^ (*p* < 0.0001); and CNFL 22.7 ± 4.0 mm/mm^2^ vs. 20.2 ± 2.2 mm/mm^2^ vs. 14.4 ± 5.2 mm/mm^2^ (*p* < 0.0001), respectively (Figure 1). For the purposes of this study, we assessed the differences between those with and without sustained abnormal corneal nerve morphology, with the latter including those with normal and temporary abnormal corneal nerve morphology.

The prevalence of DPN at baseline was 18/107 (16.8%). Of the 89 participants without DPN at baseline, 13 (14.6%) developed DPN over a median duration of 4 years (1–7 years range).

### 3.1 Association of sustained abnormal corneal nerve morphology with clinical characteristics

Participants with sustained abnormalities in CNFD (*n* = 51) were significantly older (57.2 ± 6.4 vs. 52.6 ± 9.6, *p* < 0.01) compared to those without sustained abnormalities in CNFD (n = 56) (Table 1). Those with a sustained abnormality in CNBD (*n* = 23) had a higher baseline DBP (*p* = 0.01) compared to those without sustained abnormality in CNBD (*n* = 84). Diabetes duration, HbA1c, total cholesterol, triglycerides, high-density lipoprotein (HDL), low-density lipoprotein (LDL), SBP, body weight, and BMI were comparable between the groups with and without sustained abnormal corneal nerve morphology.

**Table 1:** Comparison of clinical and neuropathic measures in patients with type 2 diabetes at baseline and their change between sustained and non-sustained abnormal corneal nerve measures.

	Non-sustained abnormal CNFD	Sustained abnormal CNFD	*P*-value	Non-sustained abnormal CNBD	Sustained abnormal CNBD	*P*-value	Non-sustained abnormal CNFL	Sustained abnormal CNFL	*P*-value
N (%)	56 (52)	51 (48)		84 (88)	23 (22)		44 (41)	63 (59)	
**Age, years**	**52.6 ± 9.6**	**57.2 ± 6.4**	**<0.01**	54.2 ± 9.0	57 ± 6.0	0.16	53.7 ± 9.6	55.5 ± 7.6	0.28
Diabetes duration, years	12.2 ± 7.1	14.5 ± 7.3	0.06	13.2 ± 7.6	13.5 ± 6.4	0.87	11.9 ± 7.7	14.2 ± 6.9	0.14
Hb1Ac baseline, %	8.3 ± 0.9	8.2 ± 1.0	0.59	8.2 ± 1.0	8.4 ± 0.9	0.43	8.2 ± 0.9	8.3 ± 1.0	0.66
**ΔHb1Ac, %**	**−0.4 ± 1.4**	**0.4 ± 2.0**	**0.01**	−0.1 ± 1.9	0.1 ± 1.2	0.28	**−0.4 ± 1.3**	**0.2 ± 2.0**	**0.05**
Total cholesterol baseline, mmol/l	4.3 ± 1.2	4.2 ± 1.1	0.71	4.3 ± 1.2	4.3 ± 1.0	0.83	4.2 ± 1.2	4.4 ± 1.1	0.45
ΔTotal cholesterol, mmol/l	−0.1 ± 1.3	−0.2 ± 1.3	0.88	−0.1 ± 1.3	−0.5 ± 1.3	0.16	−0.2 ± 1.4	−0.1 ± 1.3	0.92
Triglyceride baseline, mmol/l	1.9 ± 1.3	1.6 ± 0.9	0.21	1.8 ± 1.2	1.6 ± 0.8	0.42	1.8 ± 1.2	1.8 ± 1.0	0.97
**ΔTriglyceride, mmol/l**	**−0.3 ± 1.2**	**0.3 ± 1.3**	**<0.05**	0.0 ± 1.4	−0.2 ± 0.8	0.43	−0.2 ± 1.3	0.1 ± 1.3	0.21
HDL baseline, mmol/l	1.1 ± 0.3	1.1 ± 0.3	0.80	1.1 ± 0.3	1.1 ± 0.3	0.79	1.1 ± 0.3	1.1 ± 0.3	0.79
ΔHDL, mmol/l	0.1 ± 0.3	0.1 ± 0.3	0.50	0.1 ± 0.3	0.1 ± 0.4	0.65	0.1 ± 0.3	0.1 ± 0.3	0.79
LDL baseline, mmol/l	2.4 ± 0.9	2.4 ± 0.9	0.80	2.4 ± 0.9	2.5 ± 0.8	0.48	2.3 ± 0.9	2.5 ± 0.9	0.30
ΔLDL, mmol/l	−0.2 ± 0.9	−0.3 ± 1.0	0.73	−0.2 ± 1.0	−0.5 ± 0.8	0.22	−0.3 ± 0.9	−0.2 ± 1.0	0.75
Systolic BP baseline, mmHg	124.8 ± 17.2	128.5 ± 18.7	0.28	125.0 ± 16.6	132.5 ± 21.6	0.07	123.4 ± 16.8	128.8 ± 18.5	0.13
ΔSystolic BP, mmHg	6.2 ± 17.1	2.5 ± 21.5	0.33	5.8 ± 17.5	−0.4 ± 24.5	0.17	6.8 ± 17.1	2.8 ± 20.7	0.30
**Diastolic BP baseline, mmHg**	75.6 ± 8.7	77.6 ± 10.7	0.31	**75.3 ± 9.0**	**81.1 ± 10.9**	**0.01**	75.1 ± 8.3	77.6 ± 10.5	0.19
**ΔDiastolic BP, mmHg**	0.2 ± 11.2	−3.3 ± 11.2	0.11	**−7.0 ± 12.5**	**0.1 ± 10.4**	**<0.01**	**−3.5 ± 11.1**	**1.6 ± 10.9**	**<0.05**
Body weight baseline, kg	85.2 ± 13.3	85.1 ± 12.3	0.96	84.9 ± 12.6	85.2 ± 12.9	0.93	85.1 ± 12.7	85.2 ± 13.0	0.96
ΔBody weight, kg	−4.8 ± 14.3	−2.0 ± 7.9	0.22	−1.9 ± 5.6	−3.9 ± 12.9	0.46	−3.1 ± 13.0	−4.1 ± 9.7	0.67
BMI baseline, kg/m^2^	31.5 ± 3.7	31.9 ± 3.4	0.50	31.8 ± 3.3	31.6 ± 3.6	0.79	31.7 ± 3.4	31.6 ± 3.7	0.85
ΔBMI, kg/m^2^	−3.8 ± 9.7	−1.4 ± 6.7	0.15	−1.7 ± 7.3	−2.9 ± 8.8	0.55	−2.6 ± 8.7	−2.9 ± 8.2	0.85
**DPN baseline, n (%)**	**5/56 (8.9)**	**13/51 (25.5)**	**<0.05**	12/84 (14.3)	6/23 (26.1)	0.18	4/44 (9.1)	14/63 (22.2)	0.07
**DPN onset, n (%)**	5/51 (9.8)	8/38 (21.1)	0.14	11/72 (15.3)	2/17 (11.8)	0.71	**0/40 (0.0)**	**13/49 (26.5)**	**<0.0001**
**VPT baseline, V**	9.4 ± 5.8	9.6 ± 6.4	0.87	**8.8 ± 5.2**	**12.2 ± 8.2**	**<0.05**	9.4 ± 5.6	9.6 ± 6.4	0.89
**ΔVPT, V**	**−0.5 ± 5.6**	**3.0 ± 11.3**	**<0.05**	1.4 ± 9.5	0.5 ± 7.0	0.68	**−0.7 ± 6.0**	**2.5 ± 10.5**	**0.05**
ESC feet baseline, μS	58.2 ± 17.8	58.3 ± 16.8	0.99	58.2 ± 16.6	58.4 ± 19.8	0.97	59.5 ± 17.4	57.4 ± 17.2	0.54
ΔESC feet, μS	2.0 ± 15.7	0.0 ± 17.6	0.53	2.6 ± 16.8	−4.4 ± 15.0	0.07	4.6 ± 17.7	−1.3 ± 15.5	0.07
Burning pain and/or numbness at baseline, n (%)	31/54 (57.4)	37/51 (72.5)	0.11	51/82 (62.2)	17/23 (73.9)	0.30	23/42 (54.8)	45/63 (71.4)	0.08
**Burning pain and/or numbness at follow-up, n (%)**	**24/56 (42.9)**	**33/50 (66.0)**	**0.01**	44/84 (52.4)	13/22 (59.1)	0.57	**15/44 (34.1)**	**42/62 (67.7)**	**0.001**
**DN4 baseline, score**	**2.0 ± 2.1**	**3.0 ± 2.6**	**<0.05**	2.3 ± 2.2	3.1 ± 3.0	0.19	2.1 ± 2.3	2.8 ± 2.5	0.13
ΔDN4, score	−0.2 ± 1.6	−0.6 ± 2.4	0.33	−0.2 ± 1.8	−1.0 ± 2.6	0.07	−0.6 ± 1.4	−0.2 ± 2.4	0.25

Numeric variables are summarized as mean ± standard deviation and compared using an independent *t*-test.CNFD, corneal nerve fiber density; CNFL, corneal nerve fiber length; CNBD, corneal nerve branch density; VPT, vibration perception threshold; ESC, electrochemical skin conductance; DPN, diabetic peripheral neuropathy; Δ, differences.

The bold values indicate variables with statistically significant *p*-values.

In those with sustained abnormal CNFD, there was a progressive increase in ΔHbA1c (0.4 ± 2.0% vs. 0.4 ± 1.4%, *p* = 0.01) and triglycerides (0.3 ± 1.3 mmoL/L vs. −0.3 ± 1.2 mmoL/L, *p* < 0.05) compared to those without sustained abnormal CNFD. In those with sustained abnormal CNBD or CNFL, there was a progressive increase in Δdiastolic BP (0.1 ± 10.4 vs. −7.0 ± 12.5, *p* < 0.01, and 1.6 ± 10.9 vs. −3.5 ± 11.1, *p* < 0.05).

### 3.2 Association of sustained abnormal corneal nerve morphology with neuropathy progression

At baseline, sustained abnormal CNFD was associated with a higher prevalence of DPN (25.5% vs. 8.9%, *p* < 0.05) and neuropathic symptoms (DN4 score: 3.0 ± 2.6 vs. 2.0 ± 2.1, *p* < 0.05). Sustained abnormal CNBD was associated with higher VPT (12.2 ± 8.2 vs. 8.8 ± 5.2, *p* < 0.05). There was no association between abnormal corneal nerve morphology and sudomotor function (*p* = 0.54–0.99).

At follow-up, sustained abnormal CNFD was associated with a progressive increase in vibration perception ΔVPT (3.0 ± 11.3 vs. −0.5 ± 5.6, *p* < 0.05) and a higher percentage of patients with burning pain, numbness, or both (66.0% vs. 42.9%, *p* = 0.01). The decrease in vibration perception was associated with diabetes duration (*p* = 0.001), weight loss (*p* = 0.01), increased triglycerides (*p* < 0.0001), and decreased HDL (*p* = 0.001). After adjusting for these confounders, a sustained abnormal CNFD was associated with a 3.6 V increase in VPT (95% CI, 0.3–6.9, *p* < 0.05) over a median duration of 4 years (1–7 years range) (Table 2). Δtriglyceride levels were excluded from the multivariable regression analysis due to collinearity. Sustained abnormal CNFL was associated with the onset of DPN (26.5% vs. 0.0%, *p* < 0.0001), a higher proportion of patients with burning pain, numbness, or both (67.7% vs. 34.1%, *p* = 0.001), and an increase in vibration perception ΔVPT (2.5 ± 10.5 vs. −0.7 ± 6.0, *p* = 0.05). Sustained abnormal CNFL and CNBD were non-significantly associated with a progressive loss of sudomotor function (*p* = 0.07) and increased neuropathic symptoms (*p* = 0.07).

**Table 2:** Associations of sustained abnormal corneal nerve measures with change in neuropathy.

Dependent variable	Independent variable	Coefficient	95% CI	*P*-value
ΔVPT, V	**Sustained abnormal CNFD**	**3.5**	**0.09 to 7.0**	**<0.05**
	Sustained abnormal CNBD	−0.9	−5.2 to 3.4	0.68
	Sustained abnormal CNFL	3.2	−0.3 to 6.7	0.07
Model 1	**Sustained abnormal CNFD**	**3.6**	**0.3 to 6.9**	**<0.05**
	Diabetes duration, years	0.3	0.1 to 0.5	0.01
	ΔBody weight, kg	−0.2	−0.3 to −0.05	<0.01
	ΔHDL, mmol/l	−9.8	−15.8 to −3.8	<0.01
Model 2	Sustained abnormal CNFD	2.9	−0.4 to 6.2	0.09
	Diabetes duration, years	0.2	−0.02 to 0.5	0.07
	ΔBody weight, kg	−0.2	−0.3 to −0.01	<0.05
	ΔTriglyceride, mmol/l	1.7	0.3 to 3.1	0.01
	ΔHDL, mmol/l	−7.3	−13.6 to −1.0	<0.05
ΔDN4 questionnaire	Sustained abnormal CNFD	−0.4	−1.2 to 0.4	0.33
	Sustained abnormal CNBD	−0.9	−1.8 to 0.08	0.07
	Sustained abnormal CNFL	0.5	−0.3 to 1.3	0.25
ΔESC feet, μS	Sustained abnormal CNFD	−2.0	−8.5 to 4.4	0.53
	Sustained abnormal CNBD	−7.0	−14.7 to 0.7	0.07
	Sustained abnormal CNFL	−5.9	−12.4 to 0.6	0.07
Dependent variable	Independent variable	Odds ratio	95% CI	P-value
DPN onset	Sustained abnormal CNFD	2.5	0.7 to 8.2	0.15
	Sustained abnormal CNBD	0.7	0.2 to 3.7	0.71
	**Sustained abnormal CNFL**	**14.1**	**1.8 to 113.2**	**0.01**

CNFD, corneal nerve fiber density; CNFL, corneal nerve fiber length; CNBD, corneal nerve branch density; VPT, vibration perception threshold; ESC, electrochemical skin conductance; DPN, diabetic peripheral neuropathy; Δ, differences.

The bold values indicate variables with statistically significant *p*-values.

### 3.3 Predictive ability of abnormal baseline and sustained abnormal corneal nerve measures for diabetic peripheral neuropathy onset

**Figure 2:**
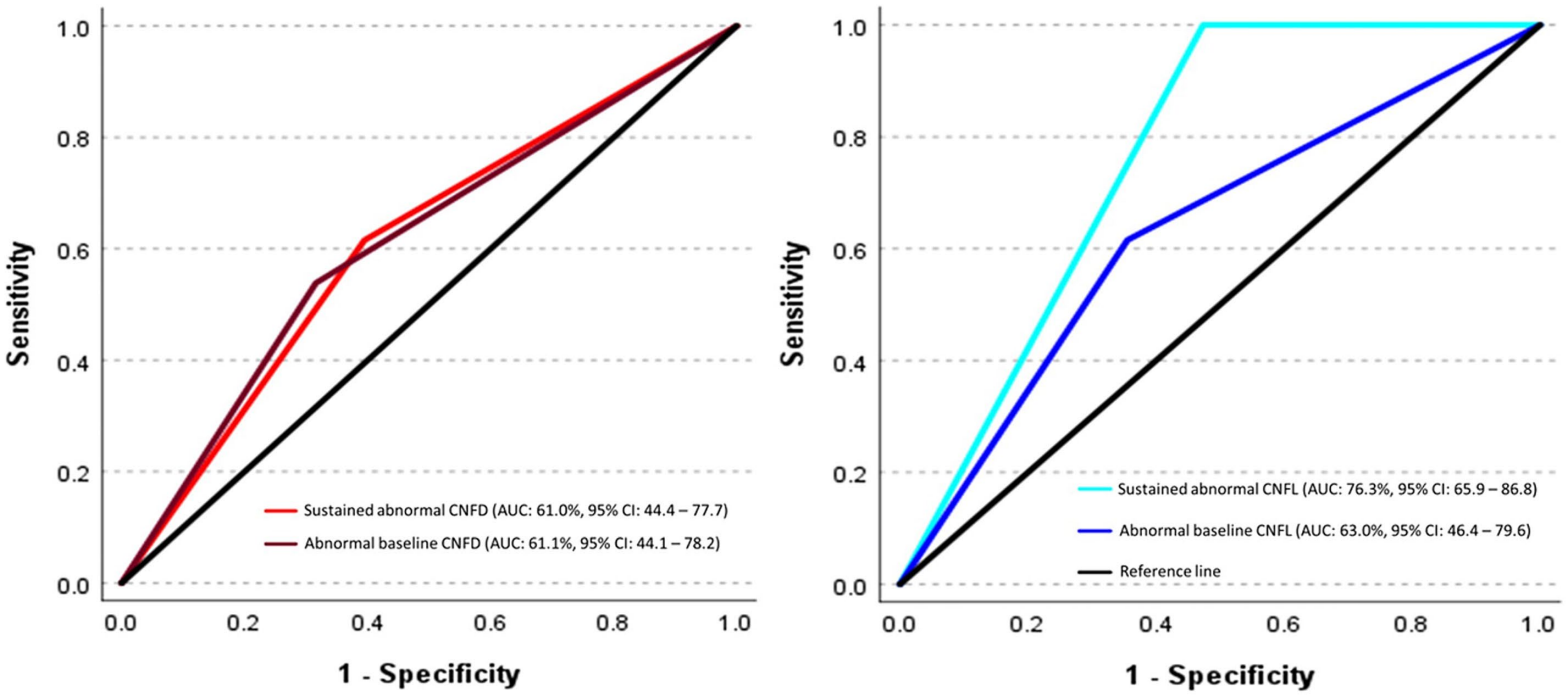
ROC curves show the predictive ability of abnormal baseline and sustained abnormal corneal nerve measures for incident diabetic peripheral neuropathy. The ROC analysis is expressed as the area under the curve (AUC) for corneal nerve fiber measures in discriminating between individuals who did and did not develop diabetic peripheral neuropathy (DPN).

Sustained abnormal CNFL was the only measure to distinguish T2D participants who did (*n* = 13) and did not (*n* = 76) develop DPN (AUC: 76.3, 95% CI: 65.9–86.8%, *p* < 0.0001) (Figure 2). Abnormal baseline CNFL (AUC: 63.0, 95% CI: 46.4–79.6%, *p* = 0.12), abnormal baseline CNFD (AUC: 61.1, 95% CI: 44.1–78.2%, *p* = 0.20), abnormal baseline CNBD (AUC: 48.6, 95% CI: 31.8–65.4%, *p* = 0.87), sustained abnormal CNFD (AUC: 61.0, 95% CI: 44.4–77.7%, *p* = 0.19), and sustained abnormal CNBD (AUC: 47.8, 95% CI: 31.1–64.5%, *p* = 0.80) did not predict the development of DPN.

## 4 Discussion

A key finding of this study is that sustained corneal nerve damage predicts the onset of DPN and the progression of neuropathic symptoms and deficits in patients with T2D. Of the three CCM measures, sustained abnormal CNFL had the highest predictive ability for DPN onset, while abnormal baseline CNFL had limited predictive capacity.

The predictive utility of CCM for incident DPN has been substantiated in two independent studies (Pritchard et al., 2015; Perkins et al., 2021). In a longitudinal diagnostic multinational consortium study (Perkins et al., 2021), the area under the ROC curve (AUC) of CNFL for incident DPN in T2D was 60–65% over 2–4 years and improved to 75% at year 6. The LANDMark study (Pritchard et al., 2015) reported an AUC of 66% (95% CI, 50–80%) for CNFL for incident DPN in type 1 diabetes (T1D) over 4 years. Both of these studies used only the baseline CNFL to predict the development of DPN. However, because more effective management of HbA1c, lipids, body weight, and physical activity can limit progressive corneal nerve damage (Ponirakis et al., 2020, 2022), this study determined whether sustained abnormal corneal nerve morphology could more reliably predict DPN development. Sustained abnormal CNFL was the only measure that predicted DPN onset with an AUC of 76.3%, while abnormal baseline CNFL had an AUC of 63%.

Corneal nerve measures at baseline were significantly lower in those with sustained corneal nerve damage, suggesting that sustained abnormalities reflect chronic irreversible neurodegeneration, while non-sustained loss of nerve fibers may reflect a capacity to regenerate, which may be relevant to the selection of patients in clinical trials of disease-modifying therapies. CNFL over 36 months of follow-up in healthy individuals remains stable (Dehghani et al., 2014), and a meta-analysis (Gad et al., 2022) showed that CNFL ranges from 16 to 21 mm/mm^2^ using manual analysis and from 12 to 17 mm/mm^2^ using automated analysis and remains stable. The average CNFL of 14.4 ± 5.2 mm/mm^2^ in those with sustained abnormal CNFL aligns closely with the previously determined optimal CNFL cutoff value of 14.1 mm/mm^2^ for incident DPN (Pritchard et al., 2015; Perkins et al., 2021), and thus, a CNFL value <14 mm/mm^2^ could be used to identify individuals at risk of developing DPN.

Corneal nerve damage is associated with the presence and severity of painful diabetic neuropathy (Kalteniece et al., 2022), and intraepidermal nerve fiber loss in skin biopsies has also been associated with DPN (Chen et al., 2015; Alam et al., 2017), but to a lesser extent with painful diabetic neuropathy (Thomas et al., 2023). In the current study, sustained corneal nerve damage was associated with an increased incidence of burning pain and numbness, or a combination of both, with only a moderate association with sudomotor dysfunction. There was a high prevalence of burning pain and numbness, consistent with previous studies showing a high prevalence of painful DPN in Qatar (37.5%) (Ponirakis et al., 2021) and in a multicenter study from the Gulf region (43.3%) (Ponirakis et al., 2022), attributed to poor glycemic control and obesity in the region. Corneal nerve loss is evident in pre-diabetes (Azmi et al., 2015) and recently diagnosed T2D (Pritchard et al., 2015; Perkins et al., 2021). However, corneal nerve measures show limited correlation with intraepidermal nerve fiber density (IENFD) and quantitative sensory testing (QST) (Ziegler et al., 2014; Gylfadottir et al., 2023) as corneal nerve degeneration and regeneration may follow different trajectories compared to intraepidermal nerves (Azmi et al., 2019) depending on the stage and severity of DPN. Indeed, we have previously shown corneal nerve regeneration occurs within 6 months of pancreas and kidney transplantation, while an improvement in IENFD and QST took over 12 months (Azmi et al., 2019). Gylfadottir et al. (2023) reported no correlation between corneal nerves and IENFD, QST, or the severity of neuropathy in patients with recently diagnosed and well-controlled T2D with minimal DPN, and Ziegler et al. also showed a low correlation between CCM measures and IENFD in a cohort of patients with a short duration of T2D (Ziegler et al., 2014).

This study reveals that age, elevated diastolic blood pressure, and higher HbA1c and triglycerides are predictors of sustained corneal nerve damage, which aligns with our previous multivariable regression analysis, which also showed that age, HbA1c, and body weight were predictors of reduced CNFL in T2D, while diabetes duration, LDL cholesterol, and triglycerides were predictors of reduced CNFL in T1D (Ferdousi et al., 2021). We have previously shown that physical inactivity and the use of medication-inducing weight gain are associated with a progressive decline in CNFD and CNFL in T2D (Ponirakis et al., 2022), and hypertension is independently associated with corneal nerve loss in T1D (Ponirakis et al., 2019). Thus, the evolution of corneal nerve damage and repair is highly dynamic and affected by multiple risk factors and ongoing treatment.

We acknowledge that the small sample size of the group with temporary corneal nerve damage may have limited its association with other neuropathy measures. Furthermore, the wide follow-up range of 1–7 years limits our ability to accurately determine the predictive utility of sustained abnormal CCM with incident DPN. However, the mean ± SD duration of follow-up was 3.9 ± 1.3 years, which is close to the median of 4 years, indicating a symmetric distribution of follow-up times without extreme outliers. The small SD also indicates consistency in the follow-up periods in the study. Approximately two-thirds of our cohort underwent follow-up assessments within 4–5 years, and this subset may be more reliable in exploring the association between sustained corneal nerve damage and the progression of neuropathic symptoms and deficits. The 1.5 SD cutoff for an abnormal CCM measure was chosen as the sample size for sustained abnormal CNFD (36% vs. 48%) and CNFL (39% vs. 59%) was smaller using the 2 SD compared to the 1.5 SD cutoff and would have reduced the power of the analyses. The wide range of the 95% CIs for the change in VPT with sustained abnormal CNFD and DPN onset with sustained abnormal CNFL may be attributed to abnormal lipids, high BMI, long duration of diabetes, a wide range of follow-up, and the subjective nature of the assessment of VPT. Further studies are needed to assess the association of sustained abnormal corneal nerves with IENFD, QST, and the severity of neuropathic symptoms.

In conclusion, this study shows that sustained loss of corneal nerves is associated with the development and progression of DPN, and sustained abnormal CNFL has the highest predictive ability for DPN onset. Regular monitoring of corneal nerve morphology may help to identify individuals at greater risk for DPN who should be targeted for risk factor reduction and may also help identify individuals who are more or less likely to respond to disease-modifying therapies in clinical trials.

## Data availability statement

Coded data from this study is available under data transfer agreement to any researcher. The corresponding author may be contacted to request access.

## Ethics statement

The studies involving humans were approved by the Weill Cornell Medicine Qatar IRB and Hamad Medical Corporation IRB. The studies were conducted in accordance with the local legislation and institutional requirements. The participants provided their written informed consent to participate in this study.

## Author contributions

GP: Writing – review & editing, Writing – original draft, Supervision, Project administration, Methodology, Investigation, Formal analysis, Data curation, Conceptualization. IA-J: Writing – review & editing, Supervision, Resources, Project administration, Investigation. EE: Writing – review & editing, Investigation, Data curation. MH: Writing – review & editing, Investigation, Formal analysis, Data curation. IP: Writing – review & editing, Investigation. HG: Writing – review & editing, Investigation. AK: Visualization, Writing – review & editing, Investigation. HZ: Writing – review & editing, Investigation. HA: Writing – review & editing, Investigation. MS: Writing – review & editing, Investigation. FFSM: Writing – review & editing, Investigation. LA: Writing – review & editing, Investigation. YD: Writing – review & editing, Investigation. AS: Writing – review & editing, Investigation. RS: Writing – review & editing, Investigation. FM: Writing – review & editing, Investigation. SA-T: Writing – review & editing, Investigation. FA: Writing – review & editing, Investigation. RH: Writing – review & editing, Investigation. SM: Writing – review & editing, Investigation. NH: Writing – review & editing, Investigation. AO: Writing – review & editing, Investigation. IS: Writing – review & editing, Investigation. ZM: Writing – review & editing, Formal analysis. MZ: Writing – review & editing, Investigation. YA-A: Writing – review & editing, Investigation. SA: Writing – review & editing, Investigation, Funding acquisition. RM: Writing – review & editing, Writing – original draft, Supervision, Resources, Methodology, Investigation, Funding acquisition, Formal analysis, Conceptualization.
